# HPV Testing from Dried Urine Spots as a Tool for Cervical Cancer Screening in Low-Income Countries

**DOI:** 10.1155/2015/283036

**Published:** 2015-06-09

**Authors:** Elena Rosanna Frati, Marianna Martinelli, Ester Fasoli, Daniela Colzani, Silvia Bianchi, Sandro Binda, Pierfranco Olivani, Elisabetta Tanzi

**Affiliations:** ^1^Department of Biomedical Sciences for Health, University of Milan, 20133 Milan, Italy; ^2^NAGA Onlus, 20136 Milan, Italy

## Abstract

Nowadays, several screening strategies are available to prevent cervical cancer, but inadequate resources, sociocultural barriers, and sampling issues impede their success in low-income countries. To overcome these issues, this study aimed to evaluate the performance of human papillomavirus (HPV) testing from dried urine spots (DUS). Eighty-eight urine samples (including 56 HPV DNA positive specimens) were spotted on filter paper, dried, and stored in paper-bags. HPV DNA was detected from the DUS after 1 week and 4 weeks of storage using a polymerase chain reaction (PCR) assay. The sensitivity, specificity, and concordance of the DUS-based HPV test were evaluated by comparing the results with those of HPV testing on fresh urine samples as the gold standard. The sensitivity of the test was 98.21% (95% CI: 90.56–99.68) for DUS stored for 1 week and 96.42% (95% CI: 87.88–99.01) for DUS stored for 4 weeks. The specificity was 100% (95% CI: 89.28–100) at both time points. The concordance between DUS and fresh urine HPV testing was “almost perfect” using the *κ* statistic. These preliminary data suggest that a DUS-based assay could bypass sociocultural barriers and sampling issues and therefore could be a suitable, effective tool for epidemiological surveillance and screening programs, especially in low-income countries.

## 1. Introduction

Cervical cancer is a relevant public health problem for women worldwide, being the third most frequent cancer and the fourth most common cause of death from cancer in women [1, 2]. Overall, the World Health Organization (WHO) estimates that there are 530,000 new cases of cervical cancer each year and more than 270,000 deaths, with 85% of deaths occurring in low- and middle-income countries [3]. All cervical cancers can be attributable to a sexually transmitted infection (STI) that is caused by the human papillomavirus (HPV). HPV infections usually clear up without any intervention within a few months after acquisition, and approximately 90% of infections clear up within two years. A small proportion of infections with certain types of HPV can persist and progress to cancer [3].

HPVs are DNA viruses that are grouped into cutaneous and mucosal types according to their infection site and further subdivided into high-risk (HR) and low-risk (LR) types, depending on their association with disease malignancy. The International Agency for Research on Cancer (IARC) has included 25 types of HPV in the high-risk clade (HR-clade) by subdividing them into three groups [4]. HPV types 16, 18, 31, 33, 35, 39, 45, 51, 52, 56, 58, and 59 are classified in group 1 as “carcinogenic to humans,” while groups 2A and 2B include the “probably carcinogenic to humans” and “possibly carcinogenic to humans” genotypes, respectively. HPV16 and HPV18 are the two most common HR types in cervical cancer, causing approximately 70% of all cases worldwide [4].

Different types of screening for cervical cancer are now available, such as the conventional cytology test (Pap test) and liquid based cytology (LBC), visual inspection with acetic acid (VIA), and HPV testing for HR-HPV from cervical brush. These types of screening require adequate financial resources, a developed infrastructure, trained labour, and surveillance mechanisms for screening, investigating, treating, and following up on targeted women [5, 6]. Moreover, women's educational levels, misconceptions, and prejudices are barriers to access and to the success of cervical cancer prevention programs in low-resource countries. Due to these difficulties, the disease is often identified in the late stage, resulting in higher rates of cervical cancer incidence and mortality [7–9].

Alternative tools that can overcome these problems could improve screening coverage and reach the female population at risk in developing countries.

The use of HPV testing on urine, a noninvasive and easy-to-collect sample, could be more attractive to women because it bypasses medical examination, as well as sociocultural and religious implications. The correlation between the detection of HPV in urine and cervical samples has been reported in several studies in the literature [10–12]. In addition, HPV DNA testing is more sensitive than cytology for detecting high-grade cervical intraepithelial neoplasia (CIN), and it would provide an automated and objective assay, which would improve quality control [13].

However, urine samples require restrictive conditions for storage and transportation, especially when samples must be transported over large distances in a warm climate. This factor is particularly relevant in low-income countries where analysis laboratories can be located far away from the rural areas where women live. An alternative method of sample collection is the use of dried urine spots (DUS), that is, a urine sample spotted on blotting paper, which allows for stabilization by drying. This approach has logistical benefits because DUS are small and easily transported and the specimens can be stored at room temperature.

DUS sample collection solves several problems associated with the sampling and storage of fresh urine samples. Therefore, the DUS approach is particularly interesting, especially in developing countries, even considering that dried spots on filter paper can be successfully used for the detection of various infectious agents, as well as metabolic and genetic diseases [14–16].

Few studies have evaluated the use of dried samples on filter paper for the detection of HPV infection, and these investigations examined exclusively cervical brush samples [17–19]. No data are presently available about the use of urine samples on this type of medium.

Thus, the aim of this study was to evaluate the performance of HPV testing from DUS and to compare the results obtained with those from paired fresh urine samples.

## 2. Materials and Methods

### 2.1. Sample Collection

Urine samples were obtained from 88 immigrant women (median age: 34 years; interquartile range (IQR): 28–43 years) who attended NAGA Onlus in Milan, Italy, between June 2012 and December 2013 and were included in a large epidemiological study on HPV and* Chlamydia trachomatis* infections [20]. All of the women provided informed consent for further anonymous research testing on the residual samples. Ethics approval was obtained from the Ethics Committee of the University of Milan, Italy.

Of the urine samples, 56/88 (63.6%) were HPV DNA positive and 32 (36.3%) were HPV DNA negative. Of the HPV DNA positive samples, 40/56 (71.4%) were sustained by single infections (24 belonging to HR-clade genotypes and 16 to LR genotypes) and 16/56 (28.6%) were caused by multiple infections (4 were caused by LR genotypes and 12 by at least 1 genotype of the HR-clade).

### 2.2. Sample Preparation

Each 400 *μ*L urine sample was subdivided into eight 50 *μ*L aliquots that were each spotted on preprinted circles on a piece of filter paper (Mascia Brunelli, Italy). The DUS filter papers were dried for 3 h and then stored in paper bags in a dry location at room temperature (RT; 25–30°C) for either 1 week or 4 weeks until the analyses took place. The analyses were carried out at the Laboratories of the Department of Biomedical Sciences for Health, University of Milan, Italy.

### 2.3. Nucleic Acids Extraction

Four preprinted circles were punched or cut out from each piece of DUS filter paper using a sterile single-cut paper-punching machine or a new sterile scalpel blade. The circles were transferred into a 1.5 mL tube containing 1 mL of NucliSENS Lysis Buffer (bioMérieux, Lyon, France) and incubated on a roller mixer for 30 minutes at RT. Then the tube was centrifuged for 15 s at 1500 ×g. The lysate, with a volume of approximately 750 *μ*L, was extracted using the commercial NucliSENS EasyMAG method (bioMérieux, Lyon, France), according to the manufacturer's instructions. The nucleic acids were eluted in 100 *μ*L of the NucliSENS elution buffer.

The concentration of the extracted DNA was evaluated using a spectrophotometer (NanoDrop 2000c, Thermo Fisher Scientific Inc., Wilmington, DE, USA). The quality of DNA was validated by detection of a 268 bp fragment of the housekeeping beta-globin gene using an in-house polymerase chain reaction (PCR) assay [21].

### 2.4. HPV DNA Detection

HPV DNA was detected using an in-house nested-PCR assay based on the amplification of a 150 bp open reading frame late gene 1 (ORF L1) fragment. The nested-PCR assay was performed using a two-step amplification to either increase sensitivity or mitigate the inhibitory effect of substances potentially present in the sample. Every PCR reaction included positive (HPV-16 positive cells, Caski) and negative (water) controls. Strict laboratory precautions and quality assurance/quality control measures were followed to avoid cross contamination and carry over PCR. ELSI_F/ELSI_R primers were used for the first cycle of amplification and GP5+/GP6+ primers were used for the nested reaction, as previously described [11, 22].

The amplification products were visualized by means of electrophoresis analysis on 2% agarose gels containing ethidium bromide (0.5 mg/mL). The amplified products were compared with molecular weight standards (DNA Molecular Weight, Marker 100, Sigma-Aldrich, St. Louis, MO, USA).

### 2.5. HPV Genotyping

HPV DNA positive DUS isolates were genotyped using INNO-LiPA HPV Genotyping Extra (Fujirebio Italia, Rome, Italy), a line probe assay based on the principle of reverse hybridization, according to the manufacturer's instructions. The resulting line patterns allowed for identification of 28 different HPV genotypes: 6, 11, 16, 18, 26, 31, 33, 35, 39, 40, 43, 44, 45, 51, 52, 53, 54, 56, 58, 59, 66, 68, 69, 70, 71, 73, 74, and 82.

### 2.6. Statistical Analysis

The sensitivity and specificity of HPV testing from DUS were evaluated in comparison to HPV testing from fresh urine samples as the gold standard. These results were presented as percentages with 95% confidence intervals (95% CIs). Proportions (95% CI) were calculated using the Wilson score model by the OpenEpi statistical program (version 3.01), which is available online [23].

To determine the proportion of agreement between DUS and fresh urine testing, Cohen's unweighted kappa (*κ*) statistic was calculated by dividing the difference between the observed proportion of agreement and the expected proportion of agreement by 1 minus the expected proportion of agreement. The concordance was defined as “poor” (*k* = 0), “slight” (0.01 ≤ *k* ≤ 0.20), “fair” (0.21 ≤ *k* ≤ 0.40), “moderate” (0.41 ≤ *k* ≤ 0.60), “substantial” (0.61 ≤ *k* ≤ 0.80), “almost perfect” (0.81 ≤ *k* < 1.00), or “perfect” (*k* = 1.00).

## 3. Results

DNA was successfully extracted from all of the DUS samples stored at RT for either 1 week or 4 weeks. The evaluated DNA concentrations ranged from 6.4 to 50.2 ng/*μ*L for 1 week of DUS storage and from 7 to 30.8 ng/*μ*L for 4 weeks of DUS storage. The housekeeping beta-globin gene was amplified from all DUS samples, confirming the suitability of the DNA extraction method.

### 3.1. Detection of HPV DNA from DUS after 1 Week or 4 Weeks of Storage

Of the 56 DUS samples prepared from HPV DNA positive fresh urine, 55/56 (98.21%, 95% CI: 90.56–99.68) tested HPV DNA positive after 1 week. In contrast, HPV DNA was detected in 54/56 (96.42%, 95% CI: 87.88–99.01) of the DUS samples after storage for 4 weeks.

The DUS sample that tested HPV DNA negative after both 1 and 4 weeks of storage was prepared from an HPV-72 (LR HPV) infected urine sample. The other DUS that tested HPV DNA negative only after 4 weeks of storage was prepared from an HPV-56 (HR HPV) infected urine sample.

All DUS prepared using HPV DNA negative urine samples tested negative for HPV DNA.

The sensitivity of the HPV DNA test in DUS at 1 and 4 weeks was 98.21% (95% CI: 90.56–99.68) and 96.42% (95% CI: 87.88–99.01), respectively. The specificity for the test was 100% (95% CI: 89.28–100) after both 1 week and 4 weeks of storage. The proportion of agreement between the DUS and fresh urine tests was “almost perfect” (*κ* statistic ≥ 0.81) (Table 1).

### 3.2. HPV Genotyping

**Figure 1:**
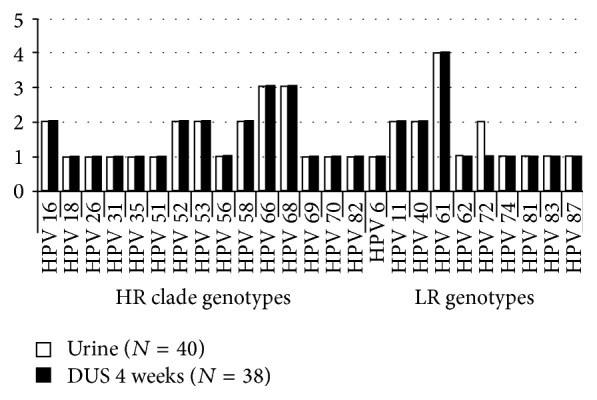
Distribution of HPV genotypes in fresh urine samples and in corresponding DUS samples after four weeks of storage.

Of the 40 DUS prepared from fresh urine samples of women infected by a single HPV genotype, 38 (95%) tested HPV DNA positive after four weeks of storage. All amplified fragments from the DUS were properly genotyped, and the distribution of HPV genotypes matched across the two sample types (Figure 1). The distribution of HR-clade and LR HPV genotypes was similar across the paired samples. In particular, 23 HPV DNA positive DUS were sustained by HR-clade genotypes and 15 were caused by LR genotypes.

## 4. Discussion

To our knowledge, this is the first report of HPV detection from urine samples stored on filter paper. The quantity and the quality of DNA extracted from DUS were comparable to those obtained by standard collection from fresh urine, as estimated by the spectrophotometric readings and the detection of the housekeeping beta-globin gene using an in-house PCR assay. Accordingly, the high molecular weight DNA extracted from DUS samples is sufficient to perform molecular assays, either traditional or high throughput.

The data obtained showed an elevated concordance between HPV DNA detection in DUS and fresh urine samples. Cohen's unweighted kappa values were very high, indicating an “almost perfect” agreement.

These preliminary data support the use of DUS as simple, rapid, and safe sampling for HPV DNA detection and genotyping by using molecular tests, such as PCR and line probe assay based on the principle of reverse hybridization. Furthermore, the use of DUS strengthens the already recognized advantages of urine samples. Collection of these noninvasive specimens is more acceptable and can bypass the ethical, social, and religious barriers of speculum exams for the collection of conventional cervical brush [7, 8, 11].

Moreover, drying urine samples on filter paper allows the DNA to be protected from degradation for a long period, as shown by the high percentage (96.4%) of HPV DNA positive samples detected after four weeks of storage at room temperature.

Due to the introduction and increasing availability of novel and powerful high-throughput molecular technologies, the optimization of methods for biological samples collection and storage has become a critical issue. These results highlight the potential use of DUS samples as an alternative means of biobanking, avoiding the high costs and logistical problems associated with the storage and transportation, especially where a cold chain is absent [19, 24]. Finally, DUS have a small size and can be easily mailed to a reference laboratory.

## 5. Conclusions

HPV testing from DUS showed an elevated sensitivity and specificity and a high concordance rate compared to HPV testing from fresh urine samples. These preliminary data suggest that a DUS-based assay could bypass sociocultural barriers and sampling issues. This approach could be a suitable and effective tool for epidemiological surveillance and screening programs, especially in low-income countries.

## Figures and Tables

**Table 1:** HPV DNA detection in fresh urine and DUS samples.

Storage time	HPV DNA	Number of fresh urine samples	Number of DUS samples	% (95% CI)	*κ* statistic
1 week	Positive	56	55	98.21 (90.56–99.68)	0.98
	Negative	32	32	100 (89.28–100)	1

4 weeks	Positive	56	54	96.42 (87.88–99.01)	0.95
	Negative	32	32	100 (89.28–100)	1

## References

[1] Ferlay J., Soerjomataram I., Ervik M. (2013). *GLOBOCAN 2012 v1.0, Cancer Incidence and Mortality Worldwide: IARC CancerBase*.

[2] Bray F., Ren J.-S., Masuyer E., Ferlay J. (2013). Global estimates of cancer prevalence for 27 sites in the adult population in 2008. *International Journal of Cancer*.

[3] Formana D., de Martel C., Lacey C. J. (2012). Global burden of human papillomavirus and related diseases. *Vaccine*.

[4] International Agency for Research on Cancer (2011). A review of human carcinogens: biological agents. *IARC Monographs on the Evaluation of Carcinogenic Risks to Humans*.

[5] Tota J. E., Ramana-Kumar A. V., El-Khatib Z., Franco E. L. (2014). The road ahead for cervical cancer prevention and control. *Current Oncology*.

[6] Dijkstra M. G., Snijders P. J. F., Arbyn M. (2014). Cervical cancer screening: on the way to a shift from cytology to full molecular screening. *Annals of Oncology*.

[7] Sankaranarayanan R., Budukh A. M., Rajkumar R. (2001). Effective screening programmes for cervical cancer in low- and middle-income developing countries. *Bulletin of the World Health Organization*.

[8] Cuzick J., Arbyn M., Sankaranarayanan R. (2008). Overview of human papillomavirus-based and other novel options for cervical cancer screening in developed and developing countries. *Vaccine*.

[9] Tornesello M. L., Rossi P. G., Buonaguro L., Buonaguro F. M. (2014). Human papillomavirus infection and cervical neoplasia among migrant women living in Italy. *Frontiers in Oncology*.

[10] Daponte A., Pournaras S., Mademtzis I. (2006). Evaluation of high-risk human papillomavirus types PCR detection in paired urine and cervical samples of women with abnormal cytology. *Journal of Clinical Virology*.

[11] Tanzi E., Bianchi S., Fasolo M. M. (2013). High performance of a new PCR-based urine assay for HPV-DNA detection and genotyping. *Journal of Medical Virology*.

[12] Ducancelle A., Legrand M. C., Pivert A. (2014). Interest of human papillomavirus DNA quantification and genotyping in paired cervical and urine samples to detect cervical lesions. *Archives of Gynecology and Obstetrics*.

[13] Kulasingam S. L., Hughes J. P., Kiviat N. B. (2002). Evaluation of human papillomavirus testing in primary screening for cervical abnormalities: comparison of sensitivity, specificity, and frequency of referral. *The Journal of the American Medical Association*.

[14] Barbi M., Binda S., Caroppo S. (2006). Diagnosis of congenital CMV infection via dried blood spots. *Reviews in Medical Virology*.

[15] Boppana S. B., Ross S. A., Novak Z. (2010). Dried blood spot real-time polymerase chain reaction assays to screen newborns for congenital cytomegalovirus infection. *JAMA—Journal of the American Medical Association*.

[16] Antunes M. V., Niederauer C. G., Linden R. (2013). Development, validation and clinical evaluation of a dried urine spot method for determination of hippuric acid and creatinine. *Clinical Biochemistry*.

[17] Banura C., Franceschi S., van Doorn L.-J., Wabwire-Mangen F., Mbidde E. K., Weiderpass E. (2008). Detection of cervical human papillomavirus infection in filter paper samples: a comparative study. *Journal of Medical Microbiology*.

[18] Alidjinou E. K., Ebatetou-Ataboho E., Sané F., Moukassa D., Dewilde A., Hober D. (2013). Cervical samples dried on filter paper and dried vaginal tampons can be useful to investigate the circulation of high-risk HPV in Congo. *Journal of Clinical Virology*.

[19] Guan Y., Gravitt P. E., Howard R. (2013). Agreement for HPV genotyping detection between self-collected specimens on a FTA cartridge and clinician-collected specimens. *Journal of Virological Methods*.

[20] Frati E., Fasoli E., Bianchi S. (2013). Prevalence of human papillomavirus and Chlamydia trachomatis infections in undocumented immigrant women in Milan, Italy. *Poster Session. EUROGIN 2013*.

[21] Puranen M., Saarikoski S., Syrjänen K., Syrjänen S. (1996). Polymerase chain reaction amplification of human papillomavirus DNA from archival, Papanicolaou-stained cervical smears. *Acta Cytologica*.

[22] Schmitt M., Dondog B., Waterboer T., Pawlita M. (2008). Homogeneous amplification of genital human alpha papillomaviruses by PCR using novel broad-spectrum GP5+ and GP6+ primers. *Journal of Clinical Microbiology*.

[23] Dean A. G., Sullivan K. M., Soe M. M. http://www.openepi.com/.

[24] Saieg M. A., Geddie W. R., Boerner S. L. (2012). The use of FTA cards for preserving unfixed cytological material for high-throughput molecular analysis. *Cancer Cytopathology*.

